# An experimental test of the effects of behavioral and immunological defenses against vectors: do they interact to protect birds from blood parasites?

**DOI:** 10.1186/1756-3305-7-104

**Published:** 2014-03-12

**Authors:** Jessica L Waite, Autumn R Henry, Jeb P Owen, Dale H Clayton

**Affiliations:** 1 Department of Biology, University of Utah, Salt Lake City, UT 84112, USA; 2 Center for Infectious Disease Dynamics and Department of Biology, The Pennsylvania State University, University Park, PA 16802, USA; 3 Department of Entomology, Washington State University, Pullman, WA 99164, USA

**Keywords:** *Haemoproteus columbae*, *Columba livia*, Hippoboscid, *Pseudolynchia canariensis*, Arthropod saliva, Defensive behavior

## Abstract

**Background:**

Blood-feeding arthropods can harm their hosts in many ways, such as through direct tissue damage and anemia, but also by distracting hosts from foraging or watching for predators. Blood-borne pathogens transmitted by arthropods can further harm the host. Thus, effective behavioral and immunological defenses against blood-feeding arthropods may provide important fitness advantages to hosts if they reduce bites, and in systems involving pathogen transmission, if they lower pathogen transmission rate.

**Methods:**

We tested whether Rock Pigeons *(Columba livia)* have effective behavioral and immunological defenses against a blood-feeding hippoboscid fly *(Pseudolynchia canariensis)* and, if so, whether the two defenses interact. The fly vectors the blood parasite *Haemoproteus columbae;* we further tested whether these defenses reduced the transmission success of blood parasites when birds were exposed to infected flies. We compared four experimental treatments in which hosts had available both purported defenses, only one of the defenses, or no defenses against the flies.

**Results:**

We found that preening and immunological defenses were each effective in decreasing the survival and reproductive success of flies. However, the two defenses were additive, rather than one defense enhancing or decreasing the effectiveness of the other defense. Neither defense reduced the prevalence of *H. columbae*, nor the intensity of infection in birds exposed to infected flies.

**Conclusions:**

Flies experience reduced fitness when maintained on hosts with immunological or preening defenses. This suggests that if vectors are given a choice among hosts, they may choose hosts that are less defended, which could impact pathogen transmission in a system where vectors can choose among hosts.

## Background

Blood-feeding arthropods and the pathogens they transmit are key contributors to the infectious disease burdens that decrease human health, agricultural productivity, and the health of wild species [1-3]. In nature, only a fraction of arthropods carry pathogens, and so vertebrate hosts are most often exposed to the bites of uninfected individuals. Yet, even if no pathogens are transmitted, blood feeding irritates and distracts hosts, often triggering behavioral defenses (such as grooming) [4,5]. With every bite, arthropods deliver salivary compounds that change the local physiological conditions at the bite site, creating an interface where the host immune system can interact with the suite of compounds in the saliva [6]. Salivary compounds may enhance acquisition of host blood by blocking hemostasis, causing vasodilation and reducing inflammation [7]. Certain compounds are potent antigens that stimulate the host immune system to act against the arthropod, e.g. causing inflammation and scabbing in response to bites [8]. This interaction between host and arthropod is a key bottleneck in the transmission of blood-borne pathogens and, as such, it is an attractive target for disease control [9].

Interactions between host and arthropod have the potential to modulate pathogen transmission. While effects of behavioral defenses on pathogen transmission have yet to be demonstrated, they have the potential to reduce pathogen transmission by preventing or reducing vector biting, or by decreasing the duration of vector blood feeding. For example, ciconiiform birds with more defensive behavior, such as foot stamping or head shaking, were better at preventing mosquitoes from feeding on them [10]. The same effect was shown for passeriform and galliform species [11]. Behavioral defenses may have indirect benefits if the energetic costs to the host associated with reducing vector fitness are offset by the benefits of smaller vector populations, and a reduced rate of pathogen transmission.

Immune defenses against blood feeding arthropods may shape host disease dynamics through interactions among the host immune system, arthropod salivary compounds, and pathogens transmitted to the host by feeding. In many cases parasites benefit from the injection of vector saliva during vector feeding, reaching higher numbers than they would by injection without saliva present [12,13]. However, if hosts are exposed to salivary compounds alone prior to parasite infection, immune responses to these compounds can have a protective effect by altering the physiological conditions at the bite (transmission) site [12].

Immune defenses against vectors may reduce pathogen transmission by interrupting the vector-induced physiological changes at the bite site that allow blood-feeding, or by indirectly reducing the overall number of vectors (through higher vector mortality and/or lower fecundity). Immune defenses against longer-term feeders, such as ticks, were discovered decades ago [14]. The idea that the immune system can also protect against shorter-term feeders (e.g. sandflies and mosquitoes) is less intuitive, but in some cases immune defenses against short-term feeders can also be effective. Immunoglobulins specific to salivary compounds can decrease feeding, acting within minutes [15]; proteolytic compounds released by basophils and eosinophils ingested with the blood meal can damage the gut of feeding vectors; these act more slowly as the meal is digested [13].

It is conceivable that different types of anti-vector defenses can interact, either enhancing the effectiveness of each (synergistic interaction), or reducing the effectiveness of each (antagonistic). Alternatively, they may simply work additively, such that combining defenses does not alter the effectiveness of each defense. The effect of anti-vector defenses in natural host-vector-pathogen systems is poorly understood. Exploring these effects can improve our understanding of the ecology and evolution of infectious disease and may suggest new avenues for disease control.

The goals of this study were to (1) test the effectiveness of host behavioral and immunological defenses against vectors, (2) determine the nature of any interaction between them, and (3) test whether these anti-vector defenses decrease the transmission (prevalence) and/or infection intensities of vector-borne parasites. We used a natural system consisting of wild caught Rock pigeons (*Columba livia*), the pigeon blood parasite *Haemoproteus columbae*, and a hippoboscid fly vector (*Pseudolynchia canariensis*) that feeds on pigeon blood.

*Haemoproteus* is the sister genus to *Plasmodium;* its life cycle resembles that of typical malaria parasites, with the exception that asexual replication takes place in the epithelial lung tissue of the vertebrate, rather than in the peripheral blood [16]. The effect of *H. columbae* on wild pigeons is chronic, leading to a gradual reduction in survival [17], with generally mild effects in captivity [18]. Sexual reproduction of *H. columbae* takes place in the fly vector, *P. canariensis*. Both male and female flies take relatively long blood meals twice daily, imbibing blood in 20–80 minute bouts [19]; both sexes transmit *Haemoproteus. H. columbae* matures to an infective stage after 10 days; these stages migrate to the salivary glands of the fly and can be transmitted when the fly bites another pigeon [20].

Typically, both fly sexes spend the majority (~70%-90%) of their time on the body of the pigeon *(pers. obs.).* Male flies will leave the bird to find a mate, and females will leave to deposit pupae on surfaces, such as the floor of the cage of a captive bird. The life cycle is unusual in that a single egg hatches *in utero*, and the subsequent three larval stages are completed inside the female before she deposits a pupated offspring. *P. canariensis* females produce one puparium every 2–3 days, once they have reached sexual maturity at about six days of age [21,22]. They usually deposit puparia in or around pigeon nests [23], but will also deposit them under the newspaper lining of pigeon cages in captivity. The flies are irritating to pigeons; infested birds double their preening activity [24].

The three specific hypotheses we tested using this system were: (1) Host behavioral and immunological defenses decrease fly fitness, specifically reducing survival and/or fecundity; (2) Host behavioral and immunological defenses interact; and (3) Host defenses against the vector reduce *H. columbae* transmission.

## Methods

### Pigeons and treatment groups

All procedures followed an animal care and use protocol approved by the University of Utah IACUC (protocols #08-08004 and #11-07018). Pigeons were bred in captivity to produce birds with no previous exposure to flies or blood parasites. All birds used in the study were bred from feral pigeon adults caught with walk-in traps in or around Salt Lake City, UT. Young pigeons, which were hatched between July 2008 and February 2009, were all mature (≥6 months old) by the start of the experiment in December 2010. Immune responses and behavioral defenses were experimentally manipulated by “priming” the immune system, or impairing preening behavior, as described below.

**Figure 1:**
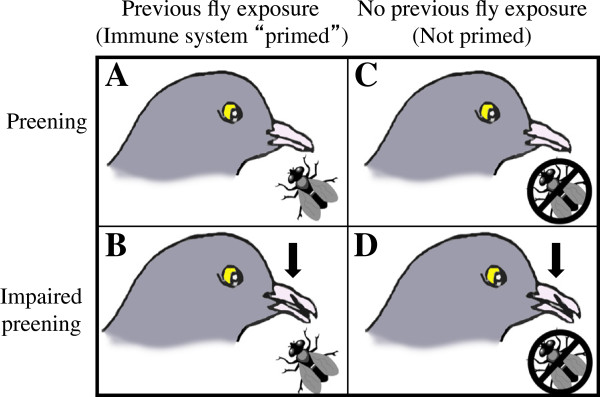
**2x2 factorial design for testing the effectiveness of behavioral and immunological defenses - and any interaction between them - against flies.** Half of the birds **(A, B)** had their immune systems primed against flies by pre-exposure to flies in a “backpack” for three weeks prior to the start of the experiment (Figure 2). The other half **(C, D)** wore backpacks with no flies. Preening was normal **(A, C)** or impaired with bits **(B, D)**.

**Figure 2:**
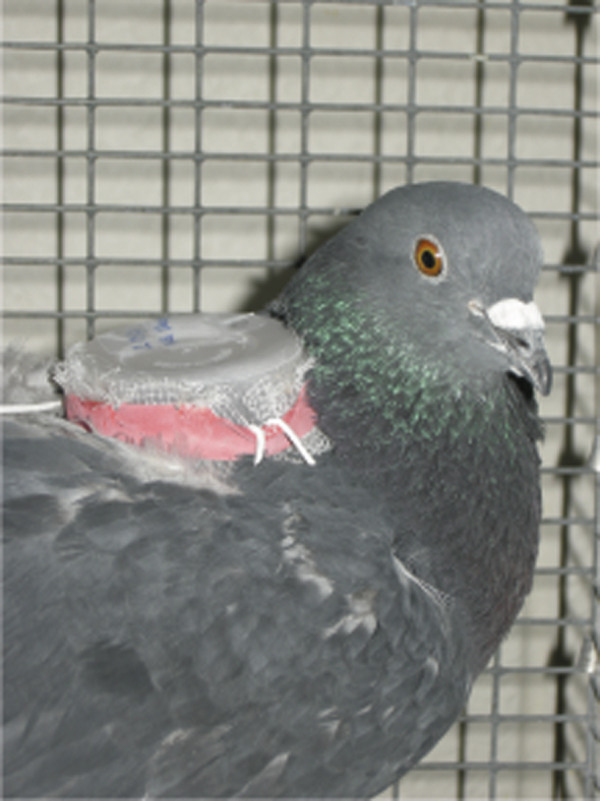
**Pigeon with a backpack, held in place by elastic straps around the wings.** Mesh netting on the bottom allowed flies to feed on the pigeon’s back, from which the feathers had been removed [26]. Pigeons could not damage or remove flies in the backpack by preening.

Pigeons had their immune systems primed (Figure 1, treatments A, B) against flies by exposing then to 10 recently eclosed flies (≤ 2 days old, unfed) in a “backpack” (Figure 2); pigeons that remained naïve to flies (C, D) had empty backpacks over this 2-week period. Feathers in the 3 cm × 3 cm region of the backpack were carefully removed to provide flies with easier access to the pigeon’s skin for feeding; feathers were also removed from control birds that wore backpacks without flies. Backpacks were removed after 2 weeks. Preening was impaired by fitting birds with harmless “bits”, which are C-shaped pieces of plastic that are inserted between the bird’s mandibles, and which spring shut in the nostrils (Figure 1, treatments B, D). Bits displace the forceps-like action of the bill required for efficient preening; they are harmless to the birds and are easy to remove [25]. The bill mandibles of preening impaired birds were trimmed weekly to prevent the mandibles from growing back to fully occlude around the bits over the 5 weeks of the experiment.

The experiment used a 2x2 factorial design (Figure 1). The experiment was replicated 12 times (N = 48 pigeons total) with treatment groups randomly assigned. When possible, sibling birds were used in the same experimental replicate with siblings placed in different treatment groups. This was done to help control for any genetic correlates of defense.

Pigeon immune system priming against flies for half of the pigeons was conducted for two weeks to allow sufficient time for a specific IgY antibody response [27]. All pigeons were fitted with fly backpacks (Figure 2) for the two weeks immediately prior to the experiment.

### Flies

Flies used in the backpacks had never been allowed to blood feed, assuring they were uninfected by *H. columbae*, which is not transovarially transmitted. Flies in the backpacks were used only for immunological priming; they were not used again later in the experiment.

**Figure 3:**
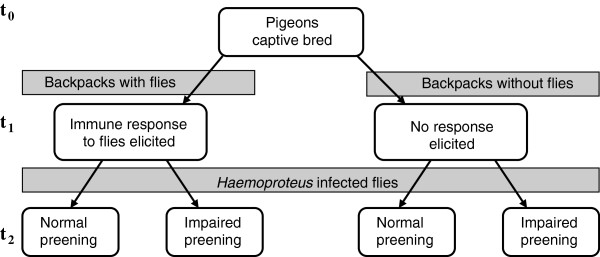
**Timeline of the experimental design.** Flies were added to pigeon backpacks in immune priming treatments at t_0_. Two weeks later (t_1_) flies and backpacks were all removed and populations of *Haemoproteus* infected flies were added to all birds. Flies were removed after 5 weeks at the end of the experiment (t_2_).

New groups of 10 freely moving flies were added to each bird in all treatments following the immunological priming period, just after backpacks were removed and birds in the preening impaired treatments had been bitted (Figure 3). Experimental pigeons were kept in cages enclosed in fly-proof netting. Each bird received five male and five female flies (sexed under a microscope at 25x magnification). Each replicate of four birds received flies from the same fly cohort. Each cohort of 40 flies was exposed to *H. columbae* parasites before flies were placed on experimental birds. For each replicate (N = 12 replicates) 40 unfed flies (≤ 2 days old) were first placed on a single naturally infected wild-caught pigeon (*H. columbae* intensity range 52 – 612, mean 273 parasites in 100 microscope fields of non-overlapping blood cells examined at 1000x, N = 12 “donor” birds, one per replicate). All cohorts of flies were left on the donor pigeon for 12 days, allowing *H. columbae* to reach the infective sporozoite stage in the fly salivary glands [28], then flies were collected from the donor birds and ten flies were transferred to each of the experimental (captive bred) pigeons. Thus, each bird received five male and five female flies of the same age with the same exposure to blood parasites across treatments within each replicate.

Dead flies and puparia were removed weekly from cages to track fly survival and reproduction; for more detailed methods see [24]. After five weeks of the experiment all flies were removed from the cages and from the birds using a combination of CO_2_ exposure for 12 minutes [29], followed by three minutes of ruffling the feathers of the birds over a white table to collect flies. The five week period was chosen because the primary goal of this experiment was to observe the effect of host defenses on fly fitness, and this time encompasses the average lifespan of an adult fly in captivity [24]. Birds were prevented from being re-infected with *H. columbae* by removing flies at this point in the experiment. The life cycle of *H. columbae* takes longer than 35 days to complete in the bird, followed by maturation to an infective stage in the fly. First parasites must develop in the bird’s peripheral blood to mature transmissible stages (typically 30–35 days) and then develop in the fly to the sporozoite stage (10–12 days) for it to be possible for a bird to be infected again by its own flies [28,30,31].

### Blood samples

Blood was sampled every three days between day 21 and day 70 of the experiment, with blood smears made each time. Smears were stained with Giemsa (diluted with buffer 1:10, pH 7.0, 50 minutes) and examined under oil immersion at 1000x for 10 minutes; if parasites were detected in a sample, then the number of parasites was quantified in 100 unique microscope fields filled with non-overlapping blood cells.

### Immunology

Additional blood samples were taken to measure IgY antibody levels in pigeon blood serum. The first blood sample was taken just prior to fitting pigeons with backpacks in order to measure baseline *P. canariensis*-specific antibody levels. Subsequent samples were taken weekly for up to 5 weeks (day 35 of the experiment). Samples were collected directly from the brachial vein into a 1.5 ml eppendorf tube, flicked 3 to 5 times to prevent large clots from forming, and immediately put on ice. Samples were spun at 10,000 rpm for 10 minutes to separate blood cells from blood serum, and the serum layer pipetted into a 1 ml O-ring sealed microcentrifuge tube, then stored at -20°C. Blood serum was analyzed using an indirect ELISA following the methods in [32].

Briefly, 96-well Nunc-MaxiSorp flat bottom ELISA plates were coated in triplicate with 100 μl of *P. canariensis* extract diluted at 1:100 in carbonate coating buffer (0.05 M, pH 9.6, Sigma). Plates were incubated for 1 hour at room temperature on an orbital table, or overnight at 4°C, and then washed five times with wash buffer (tris-buffered saline with Tween 20, Sigma). Wells were then coated with 200 μl bovine serum albumin (BSA) blocking buffer, incubated for 30 minutes at room temperature on an orbital table, and then washed five times with wash buffer. Each well was then loaded with 100 μl of pigeon serum diluted 1:100. Plates were incubated for one hour at room temperature on an orbital table and then washed five times with wash buffer. Next, 100 μl of HRP conjugated Goat-anti-Bird-IgY (Bethyl Laboratories, Inc. A140-110P) (1:5000) were added to each well, incubated at room temperature on an orbital table for one hour, and then washed five times. Finally, 100 μl of peroxidase substrate (tetramethylbenzidine, TMB: KPL Laboratories cat. 50-76-00) were added to each well. The plates were incubated for exactly 10 minutes at room temperature and the reaction was stopped using 100 μl of 2 M H_2_SO_4_ in each well, before reading optical density on a spectrophotometer using a 450-nanometer filter.

On each plate we included three wells for non-specific binding, which quantified binding of Goat-anti-Bird-IgY to the antigen. These wells received all of the reagents described above with the exception of pigeon serum. In this step, blocking buffer was used in place of serum. We also included three wells that were positive controls where a pooled sample of pigeon serum was used on all of the plates that were run so that samples could be compared across plates. We additionally included three blank wells, which received the reagents except for the antigen and serum steps, where either plain coating buffer or blocking buffer was used respectively. The mean absorbance of the non-specific binding (NSB) wells on each plate was subtracted from the absorbance measures determined above for each of the samples. Finally, we calibrated absorbance values between plates using a positive control. The reference sample absorbance was compared across all plates, and we calculated a correction factor for each plate to standardize absorbance.

### Statistical analyses

Statistical analyses were carried out using Prism v. 5.0d (GraphPad Software, Inc.). Survival analysis using a Cox proportional hazard model was run in R version 2.13.0 [33] with the survival package [34]. Because *H. columbae* is known to have different effects on male and female flies [35], fly data were analyzed by sex. To further analyze which factors influenced the overall parasite burden, we also analyzed parasitemia data in a modeling format in R [33] using the package lme4 [36].

## Results and discussion

### Effect of immune defense against flies

**Figure 4:**
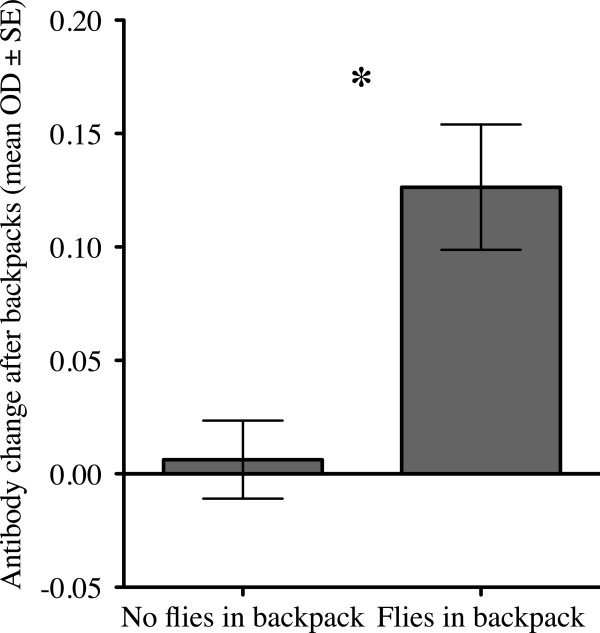
**Test of priming method.** Fly-specific IgY antibodies increased significantly in birds exposed to flies for 2 weeks compared to birds that were previously unexposed to flies (t-test, P < 0.001, see text for further statistical details). Asterisk indicates significant difference with P < 0.05. All birds were fitted with backpacks (Figure 2), half of the backpacks had ten uninfected flies to “prime” the anti-fly immune response; the other half did not have any flies.

**Figure 5:**
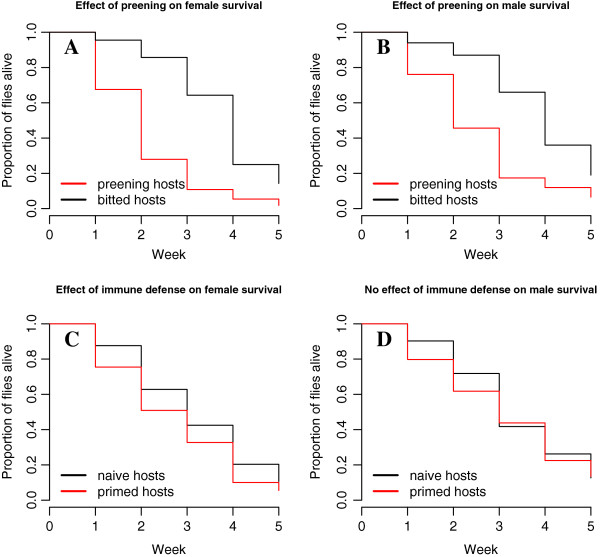
**Effects of preening and immune defenses on fly survival by sex.** Both female **(A)** and male **(B)** flies were significantly affected by preening defense (see text for statistics). However, only female flies **(C)** were affected by host immune defense over the course of 5 weeks; male flies **(D)** did not differ significantly (see text).

Immunologically “priming” pigeons against flies by exposure to uninfected flies significantly increased their anti-fly antibody levels compared to naïve controls (Figure 4; t-test, t = 3.653, df = 45, P = 0.0007). However, birds that began the experiment without such priming were able to “catch up” in their anti-fly IgY antibody levels about 2 weeks following exposure to infected flies (between time 1 and time 2; Figure 3). The latter were indistinguishable from “primed” birds after this point, indicating that they became just as immunologically responsive as primed birds (ANOVA F_3,47_ = 0.842, P = 0.478). Flies on primed birds initially were killed at a higher rate than those on birds that were not primed; however, only female flies were significantly affected (Figure 5C, D). The immune defense increased female fly mortality by 15.5% compared to female flies on birds without prior exposure to flies.

### Effect of behavioral defense against flies

Preening also had a significant negative effect on flies (Figure 5A, B). The increase in mortality rate due to preening defense was similar between the fly sexes. Females experienced a 33.8% increase in mortality due to preening and males a 48.6% increase, compared to fly mortality on preening impaired birds. Fly survival data were analyzed with both preening and immune defenses as factors. In this preening + immune model, preening and immune defenses were each effective in decreasing fly populations over the course of 5 weeks; preening increased fly mortality rate by 33.4%, and immunological priming increased fly mortality rate by 31.7% (Cox PH preening + immune model, preening P < 0.0001, exp(coef) = 0.334, immune P = 0.007, exp(coef) = 1.317). However, there was no significant interaction between preening and immune defenses on fly survival (Cox proportional hazards (PH) test for interaction of preening*immune exp(coef) = 0.763, P = 0.18). Therefore, the preening + immune model without an interaction term provided the best fit to the data.

### Defenses affect fly sexes differently

When fly sexes were analyzed separately, again there was no significant interaction between the defenses for either female or male fly survival (females; preening*immune P = 0.22, exp(coef) = 0.709; males; preening*immune P = 0.18, exp(coef) = 0.656). Analyzing the effect of defenses as factors within each fly sex revealed that female survival was significantly decreased by both defenses (Figure 5; Cox PH preening P < 0.0001, exp(coef) = 0.338, immune P = 0.0016, exp(coef) = 1.845), whereas male survival was only decreased by preening; there was no significant effect of host immune response on males (Cox PH preening P = 0.0007, exp(coef) = 0.486, immune P = 0.16, exp(coef) = 1.359).

### Effects of defenses on fly reproduction

**Figure 6:**
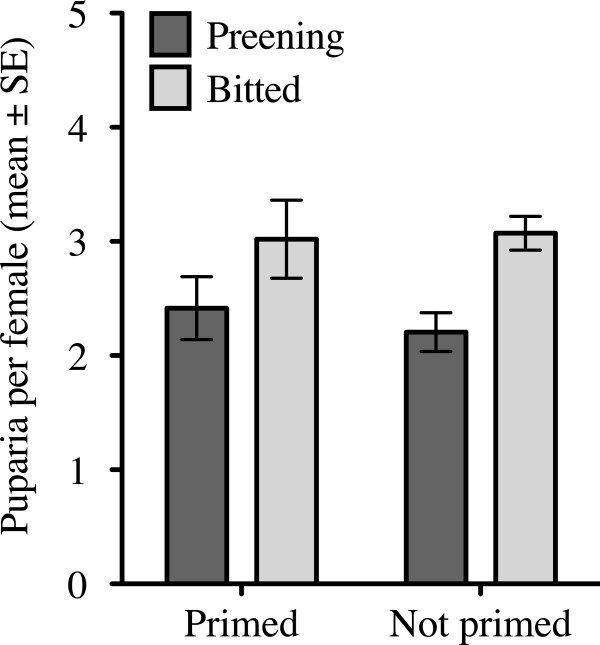
**Preening lowered female fly fecundity, but immune priming did not affect fly fecundity (see text for statistics).** None of the individual four treatment groups differed significantly from one another, although overall, preening had a significant main effect to reduce fly fecundity.

**Figure 7:**
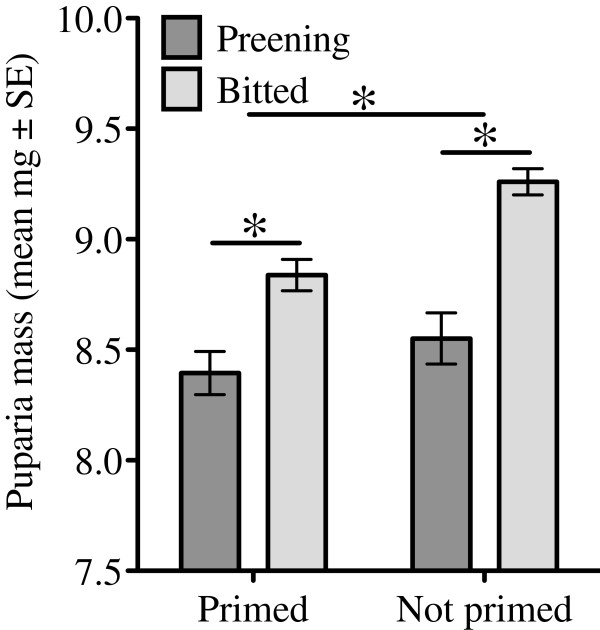
**Preening and immune defense each lowered mean puparium mass significantly (see text for statistics).** Asterisks indicate significant differences between bars with P < 0.05. The top bar represents a significant effect of immune priming on puparia mass, comparing puparia produced by flies on birds that have been immunologically primed to those that have not been primed. The lower bar represents a significant effect of preening within the immune primed group.

Female flies on preening birds also produced fewer puparia per capita than those on bitted birds over the first 2 weeks of the experiment, after which time more than 50% of the female flies were killed in the preening treatments. However, there was no significant effect of immune defense on the number of puparia, nor was there a significant interaction (Figure 6; two-way ANOVA, preening F_1,44_ = 8.876, P = 0.005, immune F_1,44_ = 0.102, P = 0.751, interaction F_1,44_ = 0.285, P = 0.597). The average mass of puparia produced during this time also differed significantly among treatments with both defenses causing lower offspring mass, but again there was no significant interaction between the defenses (Figure 7; two-way ANOVA preening F_1,962_ = 46.93, P < 0.0001, immune F_1,962_ = 11.81, P < 0.001, interaction preening*immune F_1,962_ = 2.502, P = 0.11).

### No effect of host defenses on blood parasite transmission

The impact of host defense treatment on blood parasites was analyzed using two-way ANOVAs with preening and immune response as factors. The results of these analyses are presented in Table 1. There was no significant effect of either preening or immune response on any of the parameters of blood parasite infection measured, including prevalence, peak parasitemia, rate of increase, or parasite clearance.

**Table 1:** **No influence of host anti-vector defenses on infection dynamics of the blood parasite *H. columbae***

**Measure**	**Preening defense**	**Immune defense**	**Interaction**	**Data transformation**	**Analysis**
Prevalence	P = 1.00		NA	none	Fisher’s Exact Test
Peak infection intensity	P = 0.134, F_1,44_ = 2.33	P = 1.00, F_1,44_ = 0.0	P = 0.614, F_1,44_ = 0.259	ranked	2-way ANOVA
Prepatent period	P = 0.214, F_1,43_ = 1.593	P = 0.715, F_1,43_ = 0.136	P = 0.827, F_1,43_ = 0.048	none	2-way ANOVA
Total parasites over the course of infection	P = 0.319, F_1,44_ = 1.014	P = 0.802, F_1,44_ = 0.063	P = 0.454, F_1,44_ = 0.571	ranked	2-way ANOVA
Rate of increase to peak	P = 0.079, F_1,44_ = 3.237	P = 0.610, F_1,44_ = 0.264	P = 0.798, F_1,44_ = 0.066	ranked	2-way ANOVA
Rate of clearance from peak	P = 0.135, F_1,44_ = 2.343	P = 0.801, F_1,44_ = 0.065	P = 0.801, F_1,44_ = 0.065	ranked	2-way ANOVA
% hosts that cleared the infection	P = 1.00		NA	none	Fisher’s Exact Test

(Non-normally distributed data were rank-transformed within each replicate to normalize them; rankings were compared using two-way ANOVAs).

**Figure 8:**
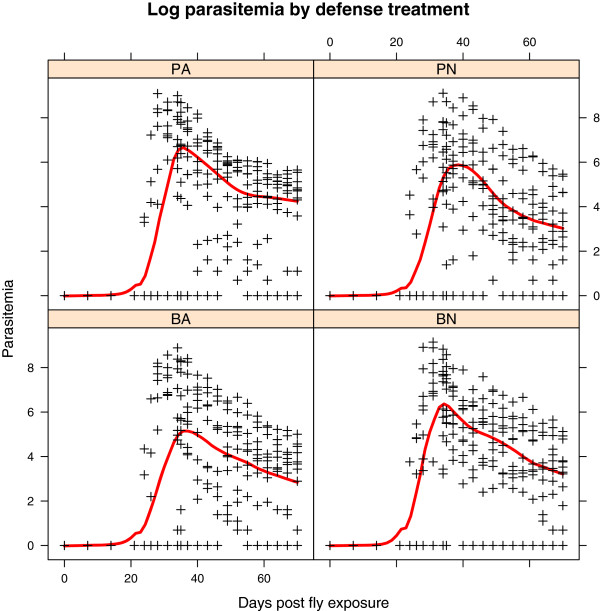
**Log blood parasite parasitemia followed a similar trajectory over time in all defense treatment groups.** Letters at the top of each square refer to treatment group: PA = preening and antibody immune response, PN = preening and naïve/no immune response, BA = bitted (no preening), and antibody immune response, BN = bitted and no immune response. The timeline for each of the plots is the same; blood was sampled repeatedly from 0 to 70 days after exposure to *Haemoproteus* infected flies.

Interestingly, parasite intensity in experimental birds was correlated with the peak parasite intensity in donor birds. Flies that were fed on more heavily infected donor pigeons transmitted blood parasites that reached greater maximum infection intensity in experimental birds, regardless of host defense treatment. However, only a small amount of the variation in experimental infection intensity was explained by donor infection intensity (r-squared = 0.1384, P = 0.009, t = 2.719, F_1,46_ = 7.392); thus birds within the same replicate had similar infection dynamics regardless of treatment group. Variation around the mean blood parasite parasitemia over time was similar across treatments; there was no effect of treatment on parasitemia (Figure 8).

Further analysis in R using a linear mixed effects model confirmed that there was no effect of defense treatment on parasitemia, even after the effect of donor infection was accounted for. The model tested included treatment day as a factor, and considered the effect of experimental replicates. The issue of autocorrelation by repeatedly sampling blood from the same individual bird was dealt with as in Pollitt *et al*. [37].

The linear mixed effects model used was the following: lme (total parasites sampled days 0–70 ~ treatment group + day as a factor + experimental replicate, method = maximum likelihood, randomized for the effect of individuals as a factor, and missing data (“na”) were excluded). (In R format: lme(totpar ~ treat + f.dpi + rep, method = “ML”, random = ~1|f.ind, na.action = na.exclude, correlation = corAR1(form = ~dpi|f.ind),data = d)).

Treatment was nonsignificant (df = 3, F = 3.848, p = 0.7996), while day of sampling and replicate had significant effects in the model (day: df = 21, F = 9.667, p <0.0001; replicate: df = 11, F = 3.391, p = 0.003).

### Discussion of effects of host defense against flies

Pigeon defenses against hippoboscid flies were effective. The previously demonstrated effectiveness of the behavioral defense of preening against flies [24] was confirmed by this study. Pigeon immune defenses were also effective against the flies, with evidence of a specific immune defense against *P. canariensis* measured as the production of anti-*P. canariensis* IgY by birds that had been immunologically “primed”. The mechanism of how the pigeon immune defense works against these mobile ectoparasites is not yet known, nor have molecules in hippoboscid fly saliva been characterized [38,39]. Other studies in related model systems suggest anti-arthropod immune responses may reduce arthropod fitness by decreasing feeding, reproduction, and survival through various mechanisms [7]. However, the use of model systems by these studies could conceivably produce results that differ from naturally co-evolved interactions [40]. Our work with a natural non-model system reinforces the results from model systems by showing that host immune defense against ectoparasites or vectors can indeed be effective.

We did not find any interaction between behavioral and immunological defenses against flies. If these defenses acted synergistically, for instance if immune defenses such as hypersensitivity responses increased itching immediately at the site of a bite, then immune defense could conceivably have directed preening behavior with the combination of defenses having a greater effect against flies than would be predicted merely by their additive effects. Delayed type hypersensitivity reactions commonly develop in vertebrates through repeated exposure to vector saliva, but they are not typically effective against vectors [41]. By itself, this does not rule out the possibility that immune responses could enhance the effectiveness of another defense, such as by directing behavioral defenses, or increasing the amount of time spent preening (not measured in this study). An alternative prediction is that defenses act antagonistically, as in the case where the cost of one defense prohibits investing fully in a second defense. Our study provided no support for the antagonistic defense hypothesis either. We found that behavioral and immunological defenses of pigeons against flies were additive, with each defense equally effective on its own or in combination.

When we examined the effectiveness of each of these host defenses against male and female flies separately, we found that preening killed both sexes; however, immune defense killed only female flies. This phenomenon may be explained by the fact that female flies take blood meals that are 40% larger than those taken by males, on average [35]. Consequently females imbibe larger quantities of immunoglobulin; they may therefore have increased their exposure to proteolytic compounds of the pigeon immune system, such as those produced by basophils and eosinophils.

In our experiment flies were fed on *H. columbae*-infected pigeons prior to being placed on birds. Previous work has shown that only female fly survival is affected by *H. columbae*, while males are unaffected, possibly due to differences in reproductive investment [35]. It is conceivable that female flies were more vulnerable than male flies to pigeon immune defenses because of the greater stress associated with blood parasite infection. The preening and immune defenses of pigeons each reduced the average fly offspring mass, a measure of the quality of offspring. However, only preening reduced the number of puparia produced by females; immune defenses had no effect on fly offspring, other than their mass. It is possible that preening behavior disrupted female feeding, and smaller blood meal sizes could have resulted in fewer and/or smaller offspring as a result.

### Why no effect of host defenses against vectors on blood parasites

In some cases previous immune experience of the host to an uninfected vector (or the vector’s saliva) provides protective immunity against pathogens, including those that cause malaria [14,42-45]. The outcome of host exposure to an infected vector might therefore be influenced by its history of exposure to that vector’s saliva. Since uninfected vectors are often more common in natural populations than infected vectors [15,41], if pre-exposure to uninfected arthropod saliva is protective, it may be a natural barrier to parasite transmission. There is some evidence for naturally acquired anti-vector immunity being protective in human populations exposed to sandfly-vectored *Leishmania*[46]. In our study, we found no evidence that pre-exposure of pigeons to uninfected hippoboscid flies afforded any protection (nor promoted any harmful effects) from *H. columbae* when birds were later exposed to populations of infected flies (Table 1). If any such effect was present, we may have been unable to detect it due to the nature of our experimental design. To track the bulk of fly lifetime survival and fecundity, infected fly populations remained on pigeons for 5 weeks. This design allowed birds to be repeatedly fed upon by infected flies for all treatment groups; thus any effect of host defense against flies would have had to be very rapid and/or very large. Any possible differences in parasitemia due to host defenses against vectors, including immune defenses from pre-exposure to fly bites, may simply have been overwhelmed. The parasite intensity of donor pigeons explained 14% of the intensity in experimental birds regardless of defense treatment, which suggests additional factors, such as parasite genetic determinants of growth or replication rate, explain some of the remaining variation in transmission dynamics [47,48].

Inherent in our experimental design was the fact that flies could not move between birds. Thus, any potential influence of pigeon infection status or defenses on host preference by flies could not be detected in this design. Hippoboscid flies have been shown to remain on *Haemoproteus* infected birds in a frigatebird-hippoboscid fly-*Haemoproteus* system compared to uninfected birds in a study of fly movement among hosts [49]. Flies may remain on infected birds if *Haemoproteus* is costly to the fly, perhaps by making the fly sick or by taking energy from the fly that might have been used for flight and movement away from the host. Alternatively, if *Haemoproteus* infected birds preen less, then they may be more attractive in having fewer defenses against flies. Indeed, now with the knowledge that anti-vector immune responses are effective against such mobile and relatively rapidly feeding hippoboscid flies, it would be very interesting to learn whether flies detect and prefer hosts without prior immune experience. Anecdotal evidence that flies prefer to feed on nestling pigeons [23] suggests that this may be the case. Vector preference for host immune defenses (or lack thereof) is a topic that has been little explored, though the potential effects of vector behavior influenced by host immune responses could have large consequences for disease transmission dynamics (unpublished observations). It would be interesting to repeat our experiment using flies that can choose which host to bite.

## Conclusions

When pigeons were exposed to hippoboscid flies they developed a specific antibody response against the flies. Populations of flies on pigeons that had anti-fly antibodies had lower fitness, including shorter average lifespan and lower offspring mass. The behavioral defense of preening was also effective against flies. Birds that could preen killed flies faster and flies also produced fewer offspring, again of lower mass than compared to birds without preening defense. The two defenses were neither synergistic nor redundant, but additive. Neither defense, nor the combination of the two defenses, reduced the transmission of blood parasites by flies in this study. In future work, allowing flies to choose between infected and uninfected hosts would provide a test of the hypothesis that vectors prefer hosts with fewer defenses.

## Competing interests

The authors declare that they have no competing interests.

## Authors’ contributions

JLW designed and conducted the study, analyzed data, and drafted the manuscript, ARH assisted with data collection, JPO and JLW designed and carried out the ELISA immunoassays, DHC and JPO assisted with designing the study and manuscript revisions. All authors read and approved the final manuscript.
